# Peabody Developmental Motor Scales-2: The Use of Rasch Analysis to Examine the Model Unidimensionality, Motor Function, and Item Difficulty

**DOI:** 10.3389/fped.2022.852732

**Published:** 2022-04-20

**Authors:** Nadia Cristina Valentini, Larissa Wagner Zanella

**Affiliations:** ^1^Human Movement Sciences Graduate Program, School of Physical Education, Physiotherapy and Dance, Universidade Federal do Rio Grande do Sul, Porto Alegre, Brazil; ^2^Department of Sports and Leisure, Instituto Federal de Educação, Ciência e Tecnologia do Rio Grande Do Sul, Sertão, Brazil

**Keywords:** validation study, Rasch analysis, PDMS-2, child development, motor assessment

## Abstract

The Peabody Developmental Motor Scales-Second Edition (PDMS-2) is a valid and reliable instrument used in several countries, including Brazil, to assess gross and fine motor skills and identify motor deficits and eligibility for intervention for children with and without disabilities. However, the analysis of PDMS-2 items regarding the unidimensionality of the model, order of item difficulty, and whether the items portray the children's developmental trajectories still lacks investigation. Therefore, this study aims to: (1) analyze the unidimensionality of PDMS-2, (2) verify the model's capacity to explain the variance in the motor function responses, and (3) identify the level of difficulty of the items for Brazilian children. Children (*n* = 637; 51% girls) newborn to 71 months (M age = 21.7, *SD* = 18.6) were assessed using the PDMS-2. The Rasch analysis was conducted; the indexes of infit and outfit, and the point-biserial correlations coefficient were analyzed. The model unidimensionality was investigated using percentages of variance in the Rasch model (40% of variance). Results indicated that (1) for reflexes subscale, 62.5% of the items had correlations with the factor above 0.60, and two items had unadjusted infit and outfit; (2) for stationary subscale, 83.3% of the correlations of the items with the factor were above 0.50, and one item had unadjusted infit and outfit; (3) for locomotion subscale, 80.0% of the correlation of the items with the factor were above 0.50; all items had adequate infit and outfit; (4) for object manipulation subscale, 79.9% of the correlation of the items with the factor were above 0.50, and one item had unadjusted infit and outfit; (5) for grasping subscale, 92.3% of the correlation of the items with the factor were above 0.50, and one item had unadjusted infit and outfit; and (6) for the visual-motor integration subscale, 73.6% of the correlation of the items with the factor were above 0.50, and six items had unadjusted infit and outfit. The items with unadjusted fit were removed for further analysis. No changes in reliability and separation of items and people scores were observed without the unadjusted items; therefore, all items were maintained. A unidimensional model was found, and the reliability and discriminant capability of the items were adequate, and all items should be used to assess children. The PDMS-2 is appropriate for assessing Brazilian children.

## Introduction

Many healthcare professionals are involved in assessing, follow-up, and providing intervention for children with disabilities, motor delays, and risk of delays (1–8). An essential aspect of assessing children with and without disabilities is to use instruments that provide pertinent information regarding developmental trajectories to assist in the intervention guidelines (7, 9–11); hence, knowing the child's functional capacity is fundamental for interpreting the assessment results. The professional's decision-making, especially concerning referrals and intervention actions, implies the accountability to select appropriate assessments that provide reliable and valid measures of child motor development.

A reliable tool used in several countries (1, 2, 4, 12–16) is Peabody Developmental Motor Scales-Second Edition (PDMS-2) (12). The PDMS-2 is a process- and product-oriented motor assessment of movement for children born up to 71 months of age. Since its inception, PDMS-2 has gone through two versions. The first version was validated in 1983 (17), and the second version was validated in 2000 (12). The first version was specially designed to detect the early onset of disorders and assess children with disabilities or delays. The second version emerged from the revision and expansion of the first version, enabling a broader, more accurate, and complete assessment of motor performance (12).

The PDMS-2 also contains items more compatible with everyday experiences, such as picking up a pencil or climbing stairs, reinforcing the scale ecological relevance. However, the analysis of the items themselves, whether they are adequate in their order of difficulty and whether they portray children's developmental trajectories, still lacks investigation. It is noteworthy that, previously, the characteristics of the item, such as the difficulty and power of discrimination for each PDMS-2 item, were examined using a two-parameter model in Item Response Theory (IRT) (12) but only for the American sample.

The use of IRT allows individual investigation of the properties of each item, estimating the difficulties, discrimination, parameters, and successes of the items. These properties of the Rasch model have led researchers to use this form of analysis to develop new assessments (18) or reevaluate instruments that lack further psychometrics evidence (1, 19, 20). Specifically, researchers have used this approach to assess the quality of items in several well-known motor assessments, for example, the Test of Infant Motor Development (18, 21), Gross Motor Function Measure (19), Bruininks-Oseretsky Test of Motor Proficiency-Second Edition (22), Child Behavior Rating Scale (20), and Assessment of Children's Hand Skills (1).

It is critical to highlight that a test's properties must be investigated repeatedly until a conclusive body of scientific evidence has been accumulated (23), allowing a trustful use of the instrument. Although the validity and reliability of the PDMS-2 have been previously examined (24), it is essential to conduct the scale items analysis to verify the unidimensionality of PDMS-2. Besides, whether the items are relevant to assess its specific construct and whether the hierarchical level of difficulty proposed in the original study with American children could be similar for the Brazilian children still need examination, especially given the importance and broad use of the instrument throughout the world. In addition, the PDMS-2 was originally developed in the United States emerged in the American culture; if each item is relevant and adequate for children from another culture is a piece of essential information with clinical repercussions. Therefore, this study aimed to analyze the unidimensionality of the PDMS-2 using the IRT, verify the model's ability to explain the variance in the motor function responses and identify the level of difficulty of the items for Brazilian children.

## Method

### Participants

Sample size estimation was conducted based on the Brazilian national data. According to the National Household Sample Survey (IBGE) (25) in 2018, the Brazilian child population was approximately 35.5 million children, including newborns to children of 12 years old. Therefore, for a 95% confidence level, Brazilian child population size (25), and a margin of error of 4%, a minimum sample size of 604 was needed to represent the national population in this study.

Consequently, in this observational and cross-sectional study, the participants were 637 children, newborns to 71 months of age. Children were attending kindergarten schools, elementary schools, or cared for at home by families. The inclusion criteria were children in the first 71 months of life, and the exclusion criteria were children with musculoskeletal disorders, genetic syndromes, and congenital malformation. All parents signed the informed consent, and the university ethical committee approved this research. The demographic data are provided in Table 1.

**Table 1:** Sample characteristics.

**Variables**	**M (SD) or *n* (%)**
Age M (DP)	21.7 (18.6)
**Gestational age**, ***n*** **(%)**	
Extremely preterm (<28 weeks)	0 (0)
Very preterm (28–32 weeks)	12 (1.9)
Moderate to late preterm (33–37 weeks)	209 (32.8)
Term (38–42 weeks)	416 (65.3)
Birth weight (grams) M (DP)	2,801 (1,211)
**Sex** ***n*** **(%)**	
Boys	312 (49)
Girls	325 (51)
**Race** ***n*** **(%)**	
White	323 (51)
Pardo	281 (44)
Black	33 (5)
**Residence** ***n*** **(%)**	
Urban	522 (82)
Rural	115 (18)
**Socioeconomic status level**, ***n*** **(%)**	
A ($ 3,426.73)	61 (10.30)
B1 ($ 1,469.89	126 (21.20)
B2 ($ 748.37)	232 (39.10)
C1 ($ 407.20)	117 (19.70)
C2 ($ 244.46)	47 (7.90)
D ($ 108.14)	10 (1.70)
**Age groups**, ***n*** **(%)**	
0–11 months	281 (44.10)
12–23 months	118 (18.50)
24–35 months	86 (13.50)
36–47 months	63 (9.90)
48–59 months	54 (8.50)
60–71 months	35 (5.50)

### Peabody Developmental Motor Scales-Second Edition

The Peabody Developmental Motor Scales-Second Edition (12) was used in this study. The instrument consists of 241 items distributed in six subscales, namely, (1) reflexes with eight items (administered to infants 0–11 months of age); (2) stationary with 30 items; (3) locomotor with 89 items; (4) object manipulation with 24 items (administered to children from 12–71 months of age); (5) grasping with 26 items; and (6) visual-motor integration with 72 items.

The PDMS-2 items reflect everyday experiences during caring and typical age-appropriate games that children enrolled in, such as rolling, crawling, and scratching a piece of paper with chalk. Items are administered according to the child's age, starting at each subscale with the definition of the child's baseline age, adequately defined through the fulfillment of the first base level performed by the child. The baseline level is obtained when the child completes three tasks with a maximum score in sequence. When the child does not perform a specific task, three attempts are offered without any visual, auditory, or verbal stimuli or facilitation. Afterward, PDMS-2 administration continues in the sequence of items up to the maximum level, defined as the level at which, in three consecutive tasks, the child obtains a score of zero; at this moment, the administration of the specific subscale is interrupted; this procedure is repeated for all subscales. Raw scores are obtained by summing the scores in each subscale and then converted in the standard scores, percentile, and z-scores. The standard score allows classifying children's motor performance into seven categories, namely, (1) very superior, (2) superior, (3) above average, (4) average, (5) below average, (6) poor, and (7) very poor.

### Procedures

The research followed the Helsinki Declaration guidelines, the university ethical committee approved research. Participants were recruited *via* contact with the school board of education, visits to early childhood schools, and social networks. We held a meeting for parents who demonstrated interest in participating (presential, phone, or social media forums), explaining the research objectives and procedures. For parents that agreed to participate, we scheduled the assessment according to the child and parents' needs. In this first meeting, parents were reinformed about all the research goals and procedures and signed informed consent. Children who speak provided verbal acceptance. Each child was individually assessed in a quiet and previously organized place. The assessments were conducted in the presence of parents or legal guardians. The administration time ranged from 45 to 60 min. If the child became unwell, tired, or tearful, the test was canceled and resumed at another time. Considering that the concentration of young children is very short, in some cases, the motor subscales were administered at different times within 5 days. Factors such as children's rest, eating, and school time were respected. Data collection was videotaped for later observation and scoring. PDMS-2 was administered according to the authors' guidelines (12) by two researchers; the leading researcher assessed all children, and the second researcher assessed 20% of the sample for interrater reliability; both researchers reassessed 20% of the videos for intrarater reliability. Both researchers were extensively trained in using the PDMS-2 before assessing the children in this study. High intrarater [intraclass correlation coefficient (ICC) > 0.97] and interrater agreement for item scores (ICC > 0.92).

### Data Analysis

Item responses and item difficulty regarding participants' performance, location of participant scores, latent trait, and items' fit indexes in the model were conducted using the Rasch analysis. The extension of the Rasch model to polytomous items and the masters' partial credit model were used (18); the scale ranged from 0 to 100, with the average difficulty of the items equaling 50. The separation index, i.e., the number of groups that can be discerned in the item hierarchy, was examined; values below 3 indicate that the variations in participants' ability and sample size were not sufficient to confirm the hierarchical difficulty of the items (26–28). For the identification of the items with unadjusted infit and outfit, we adopted recognized criteria (29); items with values near 1 are the ones that collaborate the most for the measure; values below 0.50 and between 1.50 and 2.00 do not contribute much but do not degrade the quality of the measure, and values above 2.00 represent noise or item variance not explained by the factor effect (29). Therefore, values between 0.50 and 1.50 for infit and outfit were considered adequate (29) and were adopted in the study. For the items' point-biserial correlations with the latent trail, we adopted the cutoff of above 0.30 as adequate (30).

Reliability was also examined; values below 0.30 were considered unacceptable and above 0.70 were considered acceptable. Ceiling effect was considered when more than 20% of the sample completed all the items in the scale, and floor effect was considered when more than 20% of the sample could not complete any items on the scale (28). The unidimensionality of each scale was investigated using the percentage of variance explained by the Rasch model; 40% of the variance was adopted as a strong indicator of unidimensionality (31). Residual analysis was also examined, in which the residual variance was investigated if the participants' response patterns would compose a second dimension distinct from the one-dimensional model. If a second dimension explains only 5% of the remaining variance, the one dimensionality of the scale is assumed (31). The software Winsteps 3.70 (31) and the Software R (32) were used to conduct the analyses.

## Results

### Reflexes Subscale

The PDMS-2 reflexes subscale scores' estimates, using the Rasch measurement scale, ranged from −3.14 to 1.46 with a mean of −0.48 (*SD* = 1.01). Participants' infit mean was 0.95 (*SD* = 0.41), and the outfit mean was 1.30 (*SD* = 1.49). The person separation coefficient was 1.17, and the person skill reliability estimate was 0.58.

The psychometric properties of the PDMS-2 reflexes' subscale items, included and removed, are presented in Table 2. The reliability of the subscale was 0.97 with an index of separation of 5.66; the items' infit mean was 1.00 (*SD* = 0.54), and the outfit was 1.21 (*SD* = 1.13). The items' point-biserial correlations ranged from −0.08 to 0.81 (*M* = 0.54 *SD* = 0.33), and 75% of the items had correlations with the factor above 0.30.

**Table 2:** Reflexes subscale: item difficulty, INFIT, OUTFIT, and point-biserial correlations before and after removing items.

**Reflexes subscale**								
**Item**	**All items**				**With items 1 and 2 removed**			
	**Difficulty**	**INFIT**	**OUTFIT**	**BPoint**	**Difficulty**	**INFIT**	**OUTFIT**	**BPoint**
1	1.24	1.76^*^	3.87^*^	–0.08^#^	–	–	–	–
2	0.04	1.87^*^	1.89^*^	0.22^#^	–	–	–	–
3	−1.02	1.39	1.45	0.40	−1.31	1.46	2.44	0.76
4	−0.13	0.75	0.71	0.70	0.11	1.12	1.05	0.78
5	−0.25	0.63	0.53	0.78	−0.01	0.80	0.64	0.77
6	−0.22	0.52	0.45	0.81	−0.07	0.61	0.54	0.78
7	0.04	0.51	0.40	0.78	0.43	0.57	0.32	0.76
8	0.31	0.54	0.41	0.76	0.85	0.64	0.43	0.74

*BPoint, Point-biserial correlations*.

* *Unadjusted infit or outfit value*.

# *Small correlations*.

Two items presented infit beyond what was considered acceptable. After excluding them, trustworthiness (0.64) and in the person separation coefficient (1.33) improved. This reflexes-6-item model explained 56% of the variance of the responses, supporting its unidimensionality.

**Figure 1:**
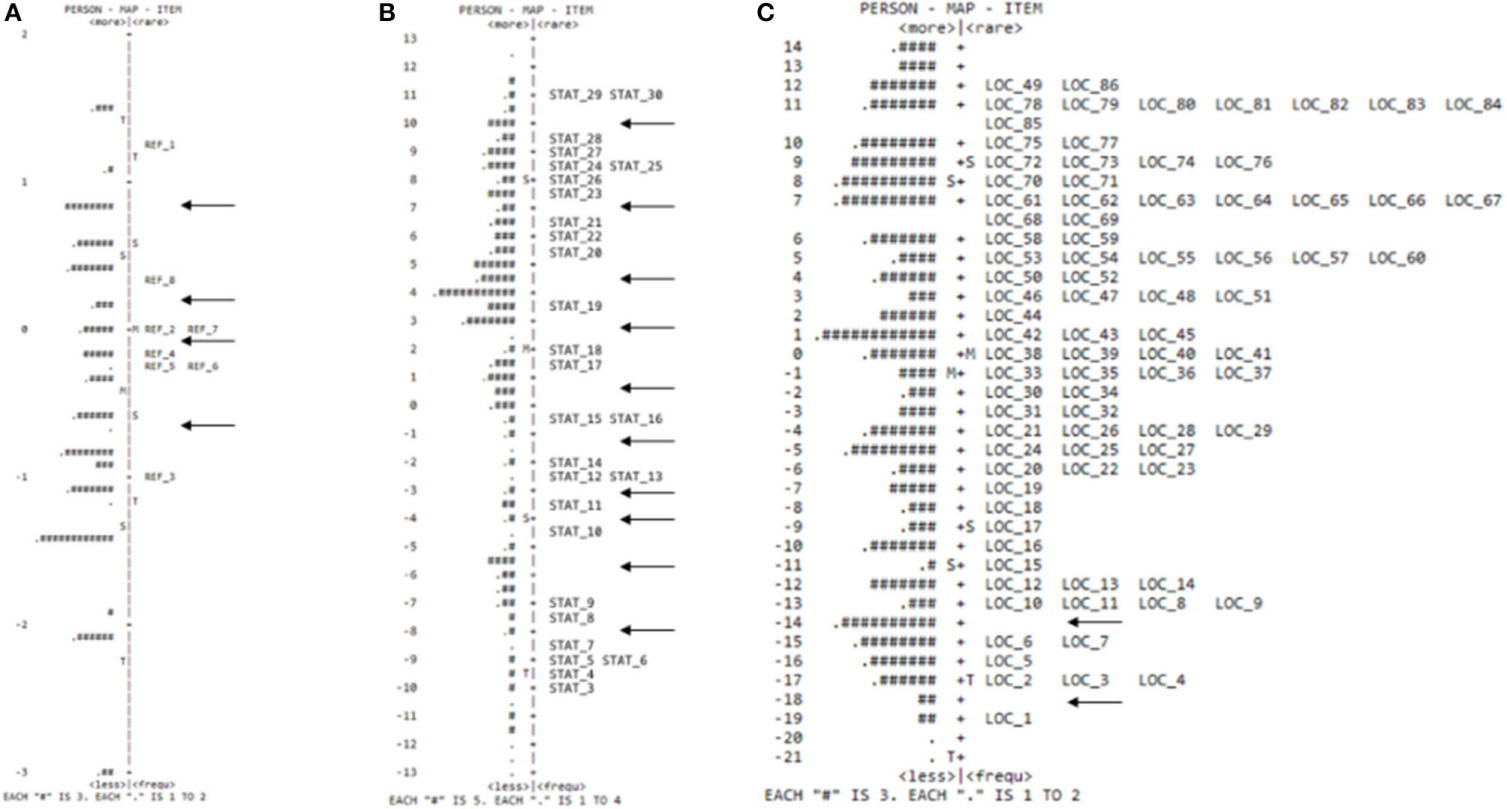
Person item map of the reflexes **(A)**, stationary **(B)**, and locomotor **(C)** subscales.

The item-person map of the PDMS-2 reflexes subscale (Figure 1A) showed that the six items were not distributed along with the entire latent trait, therefore, not covering much of the motor function distribution of the sample, also verified through the discontinuity of items, indicated by the arrows in Figure 1A.

### Stationary Subscale

The PDMS-2 stationary subscale scores' estimates, using the Rasch measurement scale, ranged from −13.29 to 12.39 (*M* = 2.01 *SD* = 6.06). Participants' infit mean was 0.91 (*SD* = 0.76), and the outfit mean was 0.62 (*SD* = 1.40). The person separation coefficient was 7.54, and the person skill reliability estimate was 0.98.

The psychometric properties of the PDMS-2 stationary subscale items, included and removed, are presented in Table 3. The reliability of the subscale was 1.00 with an index of separation of 41.80, the items' infit mean was 0.94 (*SD* = 0.31), and the outfit mean was 0.95 (*SD* = 1.61). The items' point-biserial correlations ranged from 0.48 to 0.80 (*M* = 0.65 *SD* = 0.12), with 100% of the items having correlations with factors above 0.30.

**Table 3:** Stationary subscale: item difficulty, INFIT, OUTFIT, and point-biserial correlations before and after removing items.

**Stationary subscale**								
**Item**	**All items**				**With item 1 removed**			
	**Difficulty**	**INFIT**	**OUTFIT**	**BPoint**	**Difficulty**	**INFIT**	**OUTFIT**	**BPoint**
1	−10.20	2.31^*^	0.48^*^	0.48	–	–	–	–
2	−11.04	0.75	0.10	0.43	−13.45	1.33	0.35	0.49
3	−10.09	0.89	0.14	0.51	−11.64	1.36	0.19	0.56
4	−9.74	0.56	0.09	0.52	−11.07	0.67	0.10	0.57
5	−8.88	0.51	0.08	0.55	−9.69	0.60	0.10	0.58
6	−9.04	0.86	0.12	0.47	−9.80	0.92	0.14	0.50
7	−8.53	0.97	0.71	0.45	−9.04	1.23	0.86	0.47
8	−7.53	1.02	0.29	0.54	−7.9	1.04	0.32	0.55
9	−6.80	1.02	0.16	0.58	−7.14	0.96	0.15	0.59
10	−4.41	0.86	0.22	0.76	−4.64	0.82	0.20	0.77
11	−3.59	1.08	1.65	0.77	−3.79	1.12	1.74	0.77
12	−2.36	0.58	0.23	0.79	−2.53	0.60	0.27	0.79
13	−2.54	1.07	0.38	0.67	−2.7	1.09	0.40	0.67
14	−1.78	0.66	0.36	0.66	−1.93	0.67	0.37	0.66
15	−0.68	1.00	0.55	0.65	−0.81	1.01	0.56	0.65
16	−0.35	0.98	0.65	0.65	−0.47	1.00	0.66	0.65
17	1.44	0.73	0.59	0.72	1.34	0.73	0.60	0.72
18	1.82	0.78	1.46	0.70	1.72	0.79	1.50	0.70
19	3.28	1.14	1.31	0.74	3.2	1.15	1.32	0.74
20	5.43	0.79	0.95	0.84	5.36	0.80	0.96	0.84
21	6.29	1.04	9.33	0.80	6.23	1.04	9.87	0.80
22	6.08	1.05	1.01	0.80	6.02	1.05	1.01	0.80
23	7.60	0.82	0.87	0.80	7.54	0.83	0.88	0.80
24	8.36	0.76	0.69	0.81	8.30	0.76	0.69	0.81
25	8.75	1.20	0.99	0.72	8.69	1.20	1.00	0.72
26	7.75	1.22	1.45	0.63	7.70	1.22	1.45	0.63
27	9.23	0.80	0.93	0.71	9.17	0.80	0.93	0.71
28	9.66	0.84	0.82	0.70	9.61	0.84	0.83	0.70
29	10.79	0.86	0.80	0.63	10.74	0.86	0.80	0.63
30	11.04	1.11	1.18	0.52	10.99	1.11	1.18	0.52

*BPoint: Point-biserial correlations*.

* *unadjusted infit or outfit value*.

One item had an unsatisfactory infit and was removed. After exclusion, there were no significant changes in the reliability and separation of items and persons. The variance explained by the measurement model was 81.3%, strongly indicating the stationary subscale unidimensionality.

The item-person map for the PDMS-2 stationary subscale (Figure 1B) showed that the items were distributed along with the entire latent trait continuum, covering a wide range of motor function. However, some discontinuities can be observed and were indicated by the arrows in Figure 1B.

### Locomotor Subscale

The PDMS-2 locomotor subscale scores' estimates, using the Rasch measurement scale, range from −20.77 to 13.85 (*M* = −1.54 *SD* = 9.67). Participants' infit mean was 0.88 (*SD* = 0.74), and the outfit mean was 0.47 (*SD* = 1.1). The person separation coefficient was 17.64, and the person skill reliability estimate was 1.00.

The psychometric properties of the locomotor subscale items are presented in Table 4. The scale's reliability was 1.00 with an index of separation of 47.54; the items' mean infit was 0.93 (*SD* = 0.31), and the outfit was 1.01 (*SD* = 2.24). The items' point-biserial correlations ranged from 0.20 to 0.80 (*M* = 0.58, *SD* = 0.11); 97.7% of the items had correlations with factors above 0.30. The variance explained by the measurement model was 81.9%, strongly indicating the unidimensionality of the locomotor subscale.

**Table 4:** Locomotor subscale: item difficulty, INFIT, OUTFIT, and point-biserial correlations for all items (no item was removed).

**Locomotor subscale**									
**Item**	**Difficulty**	**INFIT**	**OUTFIT**	**BPoint**	**Item**	**Difficulty**	**INFIT**	**OUTFIT**	**BPoint**
1	−19.42	0.94	0.17	0.20	44	1.81	0.94	0.33	0.72
2	−17.30	1.04	0.13	0.36	45	0.62	1.27	0.51	0.50
3	−16.78	0.73	0.08	0.40	46	2.81	0.94	0.40	0.77
4	−17.20	1.10	0.30	0.35	47	3.31	0.75	0.26	0.79
5	−15.81	0.95	0.48	0.47	48	3.11	0.61	0.10	0.77
6	−14.85	0.97	0.17	0.56	49	12.31	0.92	0.24	0.55
7	−14.69	0.96	0.32	0.56	50	3.60	0.81	0.26	0.58
8	−13.16	1.27	0.25	0.62	51	3.31	1.30	9.90	0.45
9	−12.89	0.88	0.41	0.60	52	3.60	1.22	9.90	0.48
10	−12.50	0.61	0.07	0.58	53	4.81	1.02	0.48	0.64
11	−12.65	0.82	0.09	0.45	54	4.77	1.12	9.90	0.58
12	−11.81	0.88	0.67	0.49	55	5.46	0.84	9.90	0.66
13	−11.62	1.08	0.14	0.44	56	5.25	0.94	9.90	0.58
14	−11.97	0.93	0.12	0.43	57	5.00	1.20	0.63	0.51
15	−10.79	0.83	0.38	0.48	58	5.77	0.63	0.25	0.62
16	−10.49	0.59	0.13	0.49	59	5.62	1.21	0.61	0.51
17	−9.44	0.74	0.36	0.56	60	5.29	1.23	0.51	0.52
18	−7.90	0.44	0.20	0.58	61	6.85	0.81	0.55	0.69
19	−7.14	0.92	0.53	0.61	62	6.69	1.09	0.66	0.61
20	−5.93	0.76	0.16	0.64	63	6.51	0.82	0.41	0.62
21	−3.99	0.82	0.23	0.71	64	7.21	0.90	0.59	0.67
22	−5.82	0.59	0.14	0.54	65	6.80	1.10	0.66	0.57
23	−6.00	0.55	0.10	0.47	66	6.76	0.91	0.49	0.67
24	−4.80	1.10	0.23	0.52	67	7.47	0.97	0.72	0.68
25	−4.59	0.96	0.21	0.54	68	7.02	0.95	0.55	0.61
26	−4.45	0.61	0.10	0.55	69	7.02	1.40	1.11	0.54
27	−4.72	0.85	0.14	0.52	70	7.83	1.26	1.00	0.62
28	−4.39	0.82	0.18	0.51	71	7.91	1.15	1.19	0.62
29	−4.16	1.07	0.40	0.51	72	8.97	0.67	0.45	0.80
30	−2.43	0.40	0.06	0.65	73	8.59	1.09	1.19	0.67
31	−2.54	0.73	0.20	0.65	74	9.19	1.11	0.98	0.69
32	−2.97	1.42	0.32	0.56	75	9.83	0.88	0.74	0.76
33	−0.92	0.87	0.17	0.64	76	9.23	0.92	0.79	0.70
34	−1.54	0.59	0.13	0.55	77	10.02	0.60	0.46	0.82
35	−1.31	0.62	0.16	0.54	78	10.57	0.84	0.65	0.78
36	−0.73	0.70	0.15	0.46	79	10.87	0.93	0.77	0.75
37	−0.73	1.18	0.29	0.48	80	10.73	0.97	0.82	0.67
38	−0.11	0.65	0.16	0.51	81	11.00	0.91	0.86	0.67
39	0.09	1.17	0.31	0.55	82	10.71	0.92	0.77	0.67
40	−0.37	1.02	0.28	0.52	83	11.31	0.80	0.72	0.73
41	−0.12	1.15	0.30	0.52	84	11.31	1.13	1.05	0.60
42	0.56	1.06	0.34	0.60	85	10.76	1.46	1.48	0.34
43	0.80	0.96	0.43	0.61	86	11.97	0.92	0.77	0.71

*BPoint, Point-biserial correlations*.

The item-person map for the PDMS-2 locomotor subscale (Figure 1C) showed that the items were distributed along with the entire latent trait continuum, covering a wide range of motor function.

### Object Manipulation Subscale

The PDMS-2 object manipulation subscale scores' estimates, using the Rasch measurement scale, ranged from −9.26 to 5.77 (*M* = 0.26, *SD* = 2.93). Participants' infit mean was 0.98 (*SD* = 0.55), and the outfit mean was 0.78 (*SD* = 0.84). The person separation coefficient was 4.81, and the person skill reliability estimate was 0.96.

The psychometric properties of the object manipulation subscale items, included and removed, are presented in Table 5. The scale's reliability was 1.00 with an index of separation of 47.54; the items' infit mean was 1.01 (*SD* = 0.20), and the outfit mean was 0.88 (*SD* = 0.45). The items' point- biserial correlations ranged from 0.41 to 0.79 (*M* = 0.65, *SD* = 0.12); 79.0% of the items had correlations with factors above 0.30.

**Table 5:** Object manipulation subscale: item difficulty, INFIT, OUTFIT, and point-biserial correlations before and after removing items.

**Object manipulation subscale**								
**Item**	**All items**				**With item 21 removed**			
	**Difficulty**	**INFIT**	**OUTFIT**	**BPoint**	**Difficulty**	**INFIT**	**OUTFIT**	**BPoint**
1	−6.57	1.32	0.55	0.41	−6.51	1.32	0.55	0.43
2	−6.09	1.03	0.57	0.44	−6.03	1.03	0.57	0.46
3	−4.67	0.98	0.49	0.55	−4.61	0.98	0.49	0.56
4	−3.71	0.85	0.36	0.65	−3.65	0.85	0.36	0.64
5	−3.56	0.94	0.51	0.65	−3.49	0.94	0.51	0.64
6	−2.85	1.02	0.53	0.66	−2.79	1.02	0.53	0.64
7	−2.29	0.85	0.55	0.71	−2.23	0.85	0.55	0.70
8	−1.18	1.20	0.76	0.75	−1.12	1.20	0.76	0.74
9	−1.38	0.87	0.58	0.75	−1.31	0.88	0.58	0.73
10	−0.48	1.22	1.25	0.73	−0.41	1.22	1.19	0.73
11	2.53	0.91	0.69	0.75	2.77	0.91	0.70	0.74
12	0.15	0.69	0.63	0.80	0.23	0.69	0.62	0.79
13	0.63	1.15	0.86	0.75	0.71	1.17	0.91	0.74
14	0.98	1.18	0.98	0.73	1.07	1.17	1.00	0.73
15	1.53	0.80	0.68	0.79	1.63	0.83	0.68	0.79
16	2.05	0.95	0.92	0.74	2.18	0.94	0.91	0.75
17	2.13	0.82	0.83	0.76	2.27	0.84	0.86	0.76
18	1.86	0.87	0.84	0.71	1.98	0.94	0.93	0.70
19	2.30	0.79	0.70	0.76	2.45	0.88	0.76	0.75
20	3.55	1.05	1.13	0.60	3.81	1.16	1.31	0.60
21	2.97	1.60^*^	2.07^*^	0.41	–	–	–	–
22	3.54	0.96	0.91	0.64	3.80	1.09	1.07	0.62
23	4.06	1.22	2.05	0.44	4.37	1.32	2.47	0.48
24	4.52	1.00	1.66	0.48	4.88	1.01	1.84	0.54

*BPoint, Point-biserial correlations*.

* *Unadjusted infit or outfit value*.

**Figure 2:**
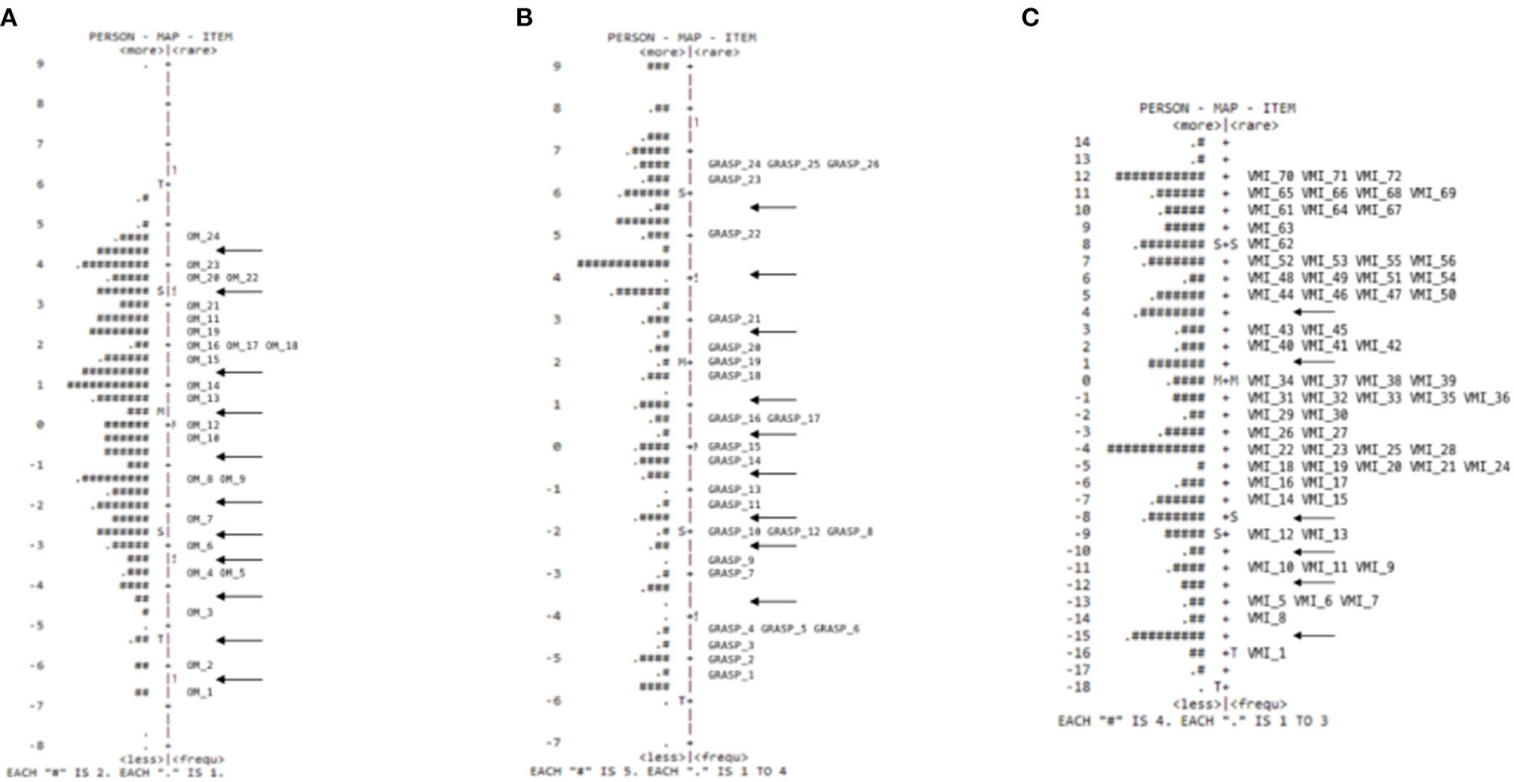
Person item map of the object manipulation **(A)**, grasping **(B)**, and visual-motor integration **(C)** subscales.

The item-person map for the PDMS-2 object manipulation subscale showed that no item exceeded the misfit values. This result also reflected the high point-biserial correlations obtained. The variance explained by the measurement model was 67.0%, strongly indicating the subscale unidimensionality. The itemperson map of the PDMS-2 object manipulation is shown in Figure 2A. The item-person map showed also for this subscale, that the items were distributed along with the entire latent trait continuum, cover a wide range of motor function.

### Grasping Subscale

The PDMS-2 grasping subscale scores' estimates, using the Rasch measurement scale, ranged from −6.76 to 9.13 (*M* = 2.21, *SD* = 4.06). Participants' infit mean was 0.88 (*SD* = 0.80), and the outfit mean was 0.63 (*SD* = 1.37). The person separation coefficient was 5.77, and the person skill reliability estimate was 0.97.

The psychometric properties of the grasping subscale item, included and removed, are presented in Table 6. The scale's reliability was 1.00 with an index of separation of 30.50. The items' infit mean was 0.94 (*SD* = 0.23), and the outfit mean was 0.84 (*SD* = 0.85). The items' point-biserial correlations ranged from 0.41 to 0.78 (*M* = 0.62, *SD* = 0.09), and 100% of the items had correlations with the factor above 0.30.

**Table 6:** Grasping subscale: item difficulty, INFIT, OUTFIT, and point-biserial correlations before and after removing items.

**Grasping subscale**								
**Item**	**All items**				**With item 1 removed**			
	**Difficulty**	**INFIT**	**OUTFIT**	**BPoint**	**Difficulty**	**INFIT**	**OUTFIT**	**BPoint**
1	−5.29	1.57^*^	1.60^*^	0.41	-	–	–	–
2	−4.85	0.85	0.48	0.53	−5.5	1.33	1.05	0.55
3	−4.67	1.03	0.35	0.53	−5.15	1.37	0.59	0.56
4	−4.46	0.61	0.20	0.58	−4.93	0.65	0.28	0.61
5	−4.37	0.89	0.48	0.54	−4.72	1.00	0.84	0.56
6	−4.48	0.68	0.15	0.48	−4.73	0.56	0.22	0.49
7	−2.87	1.04	0.28	0.59	−3.09	1.04	0.34	0.59
8	−2.03	0.94	0.50	0.66	−2.21	0.96	0.41	0.67
9	−2.57	0.72	0.25	0.59	−2.77	0.74	0.30	0.59
10	−1.92	0.64	0.14	0.56	−2.09	0.62	0.13	0.56
11	−1.21	0.93	0.24	0.60	−1.38	0.94	0.22	0.60
12	−1.88	0.97	0.24	0.49	−2.05	0.98	0.24	0.49
13	−1.04	1.26	0.52	0.58	−1.2	1.26	0.52	0.58
14	−0.28	1.25	0.68	0.65	−0.43	1.24	0.67	0.65
15	0.06	0.82	0.33	0.71	−0.09	0.81	0.32	0.71
16	0.63	0.86	0.36	0.75	0.49	0.87	0.37	0.75
17	0.70	0.88	2.48	0.67	0.55	0.88	2.47	0.68
18	1.68	0.71	0.45	0.78	1.54	0.71	0.43	0.78
19	2.00	0.85	2.07	0.72	1.86	0.85	1.91	0.73
20	2.30	1.47	2.87	0.62	2.16	1.47	2.75	0.62
21	3.00	1.08	3.23	0.66	2.86	1.09	2.91	0.66
22	5.09	0.87	1.02	0.76	4.95	0.87	1.02	0.76
23	6.42	0.84	0.63	0.71	6.29	0.84	0.64	0.71
24	6.64	0.81	0.63	0.73	6.50	0.81	0.63	0.73
25	6.62	1.01	0.94	0.66	6.49	1.01	0.94	0.66
26	6.79	0.84	0.75	0.67	6.66	0.84	0.75	0.67

*BPoint, Point-biserial correlations*.

* *Unadjusted infit or outfit value*.

Item-1 in this subscale presented infit values higher than the established as appropriate, indicating an unexpected response pattern concerning the other items. It could also be observed that this is the item with the lowest point-biserial correlation. The item is located in the lower portion of the scale and is the most accessible item to be performed by the children; therefore, it can be essential to assess children with a very low level of motor function. The Rasch model was able to explain 72% of the variance of the response patterns, which indicates the grasping scale unidimensionality.

The item-person map for the PDMS-2 grasping subscale (Figure 2B) showed that the items were distributed along with the entire latent trait continuum, covering a wide range of motor function. However, some discontinuities can be observed and were indicated by the arrows in the Figure 2B.

### Visual-Motor Integration Subscale

The PDMS-2 visual-motor integration subscale scores' estimates, using the Rasch measurement scale, ranged from −18.72 to 14.80 (*M* = −0.37, *SD* = 8.71). Participants' infit mean was 0.93 (*SD* = 0.73), and the outfit mean was 0.49 (*SD* = 1.07). The person separation coefficient was 14.59, and the person skill reliability estimate was 1.00.

The psychometric properties of the visual-motor integration subscale items, included and removed, are presented in Table 7. The scale's reliability was 1.00 with an index of separation of 45.68; the items' infit mean was 0.93 (*SD* = 0.24), and the outfit mean was 1.04 (*SD* = 2.19). The items' point-biserial correlations ranged from 0.18 to 078 (*M* = 0.59, *SD* = 0.12); 73.6% of the items had correlations with the factor above 0.50.

**Table 7:** Visual-motor integration: item difficulty, INFIT, OUTFIT, and point-biserial correlations before and after removing items.

	**Visual-motor integration subscale**																
**Item**	**All items**				**With items 3–4, 57–60 removed**				**Item**	**All items**				**With items 3–4, 57–60 removed**			
	**Difficulty**	**INFIT**	**OUTFIT**	**BPoint**	**Difficulty**	**INFIT**	**OUTFIT**	**BPoint**		**Difficulty**	**INFIT**	**OUTFIT**	**BPoint**	**Difficulty**	**INFIT**	**OUTFIT**	**BPoint**
1	−16.20	0.79	0.07	0.34	−21.16	0.48	0.03	0.43	31	−1.04	0.97	0.25	0.68	−0.9	1.04	0.27	0.69
2	−16.53	0.82	0.09	0.34	−22.22	1.47	0.26	0.42	32	−1.26	1.13	0.27	0.64	−1.14	1.23	0.28	0.65
3	−14.45	1.61^*^	0.37^*^	0.48	–	–	–	–	33	−1.10	0.85	0.17	0.59	−0.95	0.91	0.18	0.60
4	−14.90	0.89^*^	2.41^*^	0.43	–	–	–	–	34	−0.36	0.65	0.17	0.62	−0.15	0.69	0.17	0.62
5	−12.95	0.57	0.07	0.58	−13.98	1.02	0.10	0.63	35	−0.71	0.62	0.12	0.51	−0.52	0.65	0.12	0.51
6	−13.19	0.60	0.34	0.53	−14.33	0.73	0.91	0.58	36	−0.69	0.81	0.14	0.44	−0.49	0.85	0.15	0.44
7	−13.35	0.73	0.46	0.45	−14.7	1.19	1.42	0.50	37	−0.30	0.85	0.23	0.49	−0.07	0.90	0.24	0.50
8	−13.95	0.78	0.34	0.18	−14.84	0.56	0.46	0.19	38	0.49	1.41	0.48	0.54	0.76	1.50	0.54	0.54
9	−11.41	0.61	0.32	0.42	−12.11	0.63	0.39	0.42	39	0.24	1.14	0.25	0.48	0.50	1.20	0.27	0.49
10	−10.83	0.62	0.07	0.46	−11.46	0.69	0.07	0.46	40	2.01	0.55	0.17	0.70	2.39	0.60	0.18	0.71
11	−10.50	1.03	0.57	0.45	−11.11	1.12	0.59	0.45	41	2.19	0.80	0.35	0.74	2.61	0.85	0.37	0.75
12	−9.13	0.84	0.14	0.49	−9.59	0.83	0.14	0.49	42	2.17	1.16	0.36	0.66	2.58	1.28	0.40	0.67
13	−8.63	0.99	0.63	0.53	−9.07	1.03	0.21	0.53	43	3.39	0.80	0.45	0.73	3.91	0.86	0.51	0.74
14	−7.44	0.82	0.22	0.61	−7.84	0.85	0.25	0.61	44	4.82	1.34	0.98	0.79	5.46	1.46	1.20	0.80
15	−6.84	1.15	0.61	0.63	−7.2	1.18	0.61	0.63	45	3.37	0.83	0.97	0.66	3.91	0.88	1.13	0.67
16	−5.82	1.06	0.34	0.67	−6.12	1.14	0.36	0.67	46	4.82	0.79	0.50	0.75	5.46	0.85	0.59	0.76
17	−6.01	1.38	0.46	0.59	−6.32	1.37	0.46	0.60	47	5.23	0.79	0.35	0.77	5.91	0.85	0.38	0.78
18	−5.06	0.64	0.15	0.65	−5.29	0.61	0.14	0.65	48	6.04	0.90	0.40	0.81	6.78	0.95	0.43	0.82
19	−4.81	0.62	0.14	0.61	−5	0.59	0.13	0.62	49	6.37	0.82	0.35	0.78	7.14	0.82	0.32	0.80
20	−4.71	0.43	0.10	0.58	−4.84	0.44	0.10	0.58	50	5.34	0.81	0.35	0.68	6.06	0.85	0.34	0.70
21	−4.51	0.77	0.20	0.49	−4.61	0.79	0.22	0.49	51	5.67	1.19	1.18	0.57	6.42	1.22	1.20	0.59
22	−4.27	0.94	0.23	0.55	−4.35	0.98	0.24	0.55	52	6.66	1.20	0.57	0.65	7.46	1.21	0.50	0.68
23	−3.55	1.25	0.30	0.55	−3.61	1.32	0.32	0.55	53	6.81	1.09	0.78	0.66	7.63	1.10	0.79	0.68
24	−4.98	1.16	0.28	0.49	−5.06	1.21	0.29	0.50	54	6.33	0.69	0.57	0.63	7.12	0.68	0.58	0.65
25	−3.56	0.88	0.23	0.58	−3.61	0.92	0.24	0.58	55	7.23	1.36	0.83	0.61	8.09	1.56	0.99	0.62
26	−2.96	0.63	0.17	0.64	−2.99	0.66	0.18	0.64	56	6.61	0.77	0.97	0.53	7.41	0.71	1.46	0.55
27	−2.60	0.58	0.22	0.67	−2.6	0.61	0.23	0.67	57	8.29	1.23^*^	9.90^*^	0.64	–	–	–	–
28	−3.53	0.88	0.25	0.52	−3.58	0.91	0.28	0.52	58	6.76	0.98^*^	9.90^*^	0.49	–	–	–	–
29	−1.77	1.11	0.32	0.68	−1.68	1.18	0.33	0.69	59	8.91	1.02^*^	9.90^*^	0.69	–	–	–	–
30	−1.78	1.13	0.30	0.64	−1.7	1.20	0.31	0.65	60	9.32	1.02^*^	9.90^*^	0.67	–	–	–	–
61	9.69	0.76	0.62	0.83	10.65	0.84	0.77	0.81	67	10.22	1.06	1.03	0.56	11.15	0.98	0.95	0.59
62	7.86	1.19	1.77	0.49	8.59	1.35	1.99	0.51	68	11.39	0.85	0.86	0.63	12.33	0.91	0.90	0.60
63	8.79	1.20	1.07	0.59	9.63	1.24	1.12	0.60	69	11.25	1.42	1.41	0.39	12.20	1.31	1.34	0.41
64	10.35	0.81	0.73	0.76	11.27	0.80	0.73	0.76	70	11.55	0.97	0.93	0.58	12.49	0.89	0.81	0.61
65	11.29	0.84	0.76	0.72	12.23	0.90	0.80	0.69	71	11.92	1.20	1.43	0.44	12.85	1.16	1.30	0.44
66	10.54	1.09	0.96	0.68	11.47	1.04	0.88	0.69	72	11.78	0.92	0.85	0.59	12.72	0.85	0.77	0.61

*BPoint, Point-biserial correlations*.

* *Unadjusted infit or outfit value*.

In this visual-motor subscale, three types of response patterns were observed. Item-3 presented infit values higher than appropriate (1.61), indicating an unexpected response pattern concerning the other items. Item-1 and Item-2 had low outfit values (below 0.50), indicating that the observations were very predictable (28). From Item-57 to Item-60, very high outfit values were observed (9.99), indicating unexpected response patterns in the far portion of the item's difficulty. For example, Item-57 has an estimated difficulty of 8.29, whereas most estimates range from −4 to 5. Figure 2C showed that children with low motor function (in which “0” answers are expected in the item) had scores of 2, indicating that the item is too easy for a child to perform, but it is located in the more difficult part of the scale.

Such an unexpected response occurred far from the informational part of the item, which illustrates the effect of a high outfit (e.g., a hit by chance). After excluding the six items (Item-3 and Item-43 due to infit; Item-57, Item-58, Item-59, and Item-60 due to high outfit), the model explained 72% of the responses' variance. The item-person map for the visual-motor integration subscale (Figure 2C) showed that most items covered the entire range of participants' motor function distribution, correctly discriminating participants with different skill levels. In addition, the exclusion of the 6 items did not reduce the precision of the scale. Discontinuities can be observed, indicated by the arrows, in the Figure 2C.

Some isolated outfits values were slight outside the acceptable range (between 0.50 and 1.50); however, when identified alone, these non-standard values do not affect the measure since the other parameters for those items were adequate. Therefore, these items were not removed for further analysis, as they did not threaten the scale.

## Discussion

In this study, we analyzed the reliability, unidimensionality, hierarchy of items, and the levels of difficulty of the PDMS-2 in a Brazilian sample of children. The PDMS-2 is a widely used assessment to monitor child motor development and provide insights into intervention (4, 15, 16, 33, 34); its relevance for children's development requires the examination of its psychometrics across different cultures. This study was the first to use the Rasch model to examine model unidimensionality and the fit of the items in all age groups (zero to 71 months); the use of this procedure allows for a better understanding of the variance in the children's responses. Besides, the person-item map presented the item's distribution in the latent trail, its hierarchy, and the discontinuity in motor function. With this statistical procedure, the estimation of the latent trait takes into account the responses given by children and the properties of the items within the assessment (33, 34). The model analysis is based on the local independence and unidimensionality of the items, strongly associated with each other (23, 34). The basic assumption was to verify the adequate trend of response patterns; how good was the instrument to measure the individuals' latent traits. Ceiling and floor effects was also observed by percentage and frequency of responses.

The overall results showed that all the subscales were unidimensional, and for all subscales, some discontinuity in motor function and breaks in items' hierarchical order was observed. It is important to note that the few items that presented misfits were due to high values in the outfit for all subscales. The outfit represents a heightened sensitivity to unexpected responses made by children when performing items that are too easy, below their motor capabilities, or too hard, above their motor capabilities. This result indicates that for some items, the children with low-motor function levels can perform the item correctly, and for some items, even the high-skilled children could not perform the item; the discriminant power of those few items is low. Therefore, the type of misfit observed in this study was related to random hits, i.e., low-motor function children who randomly hit difficult items, or random error, i.e., high-motor function children missed an effortless item. However, no floor or ceiling effect was observed in the sample; this means that the items were able to assess individuals with high and low ability, not requiring the addition of more accessible or more complex items to the instrument.

### Person-Item Map

The item-person map showed that for the reflexes subscale, the items were not distributed across the entire latent trait, not covering much of the distribution of sample participants. This result indicates the presence of very easy items, suitable only for assessing young babies, and intermediate items, suitable for accurately differentiating participants with average motor function. As for high-motor function children, who have already inhibited reflexes, the reflexes subscale would no longer be appropriate; the assessment of rudimentary movement is indicated despite the child's age. Reflexes are bodily reactions in response to stimuli, and of an involuntary nature, these primitive reflexes disappear as the cortex develops and the baby acquires more sophisticated motor acquisitions. For example, gait reflex was one of the items that presented a biserial correlation and negative factor loading, indicating that the increase in the participant's motor skill tends to choose the lower response categories of the item (category 0 endorsement). This skill is inhibited near 3 months of age, so the non-observation of this item during the assessment indicates that voluntary skills are prevailing against reflective items, with advancing in age and the development of the upper cortex. However, despite the rapid change in motor function in the early months of life, there is a need to assess children's reflex since it is a relevant marker of child development (12); babies with reflexes' absence or prevalence beyond the expected age need further assessment by professionals since it is an indication of possible neurological disorders (33, 34).

For the stationary, object manipulation and visual-motor integration subscales, all or the majority items cover the entire range of the participants' performance distribution, correctly discriminating children with different levels of motor function. For the locomotor subscale, no item exceeded the misfit values, although Item-30 that assesses the child's ability to stand had a very low infit and outfit values, such as values, despite not being constructive for the scale, that do not lessen the subscale validity (23). We also found that for the grasping subscale, the items covered a good part of the range of the skill; however, in the mid- and lower mid-point of the scale, the addition of which could improve the subscale motor function accuracy.

### Model's Unidimensionality

Overall, the results indicated that most of the PDMS-2 items on the subscales assessed the intended constructs and that the subscales were unidimensional. However, there are some inconsistencies regarding the subscale items adjustments, mainly in two items for the reflexes subscale, one for the stationary, one for object manipulation, one for grasping, and six for the visual-motor integration subscale. We examined if excluding items with misfits would improve the scale indexes; if the reliability improved, those items did not contribute to the individual subscales or the PDMS-2 global constructs. However, it was verified that removing the non-adjusted items from the scale did not influence its structure and indexes result. Therefore, those are items that should be maintained to assess children; however, their capacity to discern different levels of performance is less relevant than the other items; caution is recommended in the interpretation of those items. It is possible that these items may measure a different construct or had a confounding factor's effect (e.g., movement experience), a plausible explanation for the inadequacy of the items on the subscales.

For example, a child who has had little or no experience playing with cords will have difficulties passing the cord through a six-hole strip may not perform Item-58 of visual-motor integration (i.e., putting the cord) consistently. In contrast, other children whose parents support safe care autonomy could be familiar with the task since they must deal with their tennis shoes daily. Another possible explanation is related to item challenge; the items perceived as more difficult or easy to manage might be identified as inappropriate depending upon each child's level of motor function. We observed that some children did not comply with the examiner's demonstration and verbal instructions about Item-57 of visual-motor integration (i.e., cutting a paper by dividing it into two parts). Younger children tended to cut out a corner of the paper almost accidentally, whereas older children may perceive it as a less challenging demand and lack attention; these behaviors were often observed and may have contributed to the item's maladjustment.

Our results suggested marginal influence on the model with removing the unadjusted items; it did not change model strength; therefore, all items should be used to assess children. Although we analyzed the model fit indices with and without removing unadjusted items, it was not the goal of this study to change or adapt the scale. However, the results provided professionals who administer the scale information regarding unexpected results in some items, and therefore, caution is recommended in interpreting those items with unexpected responses (i.e., improper fit). In addition, it is essential to emphasize that the scale was not modified during its administration in the Brazilian sample; that is, the administration strictly followed all the manual guidelines. The removal of items occurs only in the data analysis. Finally, although we have examined the scale with and without poorly fitting items, the scale psychometrics remains strong in the statistical analysis even with several withdrawals of items.

### Internal Consistency and Item Discriminating Capacity

Good internal consistency indices were found through the reliability of the Rasch analysis. The presence of items that are quite easy for younger children to perform in the stationary, locomotor, object control, and visual-motor integration subscales has clinical and psychometric implications; it does not harm the subscales' strength and indicates the PDMS-2 has items with the ability to identify substantial motor delays.

The item-person map for the subscales indicated a discontinuity in the level of difficulty or the development of tasks, observed less frequently in the locomotion subscale. The items showed continuity in the locomotion subscale (Figure 1C). It can be inferred that locomotion skills, unlike object control skills, grasping, and visuo-motor integration, present a more natural sequence of development since children do not need to control any equipment for execution. The items in several subscales do not follow a continuity sequence from easy-to-difficult items; the map indicates that the subscales have an easy level item and next to a more challenging level item—it can be detected by the discontinuity between items in the figures (indicated with the arrows)—for example, Item-13 and Item-14 in the visual-motor integration subscale. Item-13 (arm extension that assesses whether the baby, in the supine position, extends one arm toward the rattle while the other arm remains at rest) and 14 (retention cubes that assess whether the baby, in a sitting position, holds the second cube in his hand and retain the two cubes for 5 s) have a different level of challenge. The difference in the level of difficulty between these two tasks, both recommended to assess a 6-month-old child, is notable since in Item-13, the child has body support from the trunk, whereas Item-14 demands great postural control with antigravity action to keep balanced in the seated position and meet the complex demand of holding a cube in each hand. The authors suggested that this alternation of difficulty between items is understood as a form of performance discrimination between children with greater or lesser motor performance. However, our results suggested that these jumps observed in item difficulty levels could be mitigated with items of intermediate difficulty.

Another interesting result in this study is regarding the item scoring system (0 = the child cannot or will not attempt the item or the attempt does not show that the skill is emerging; 1 = the child's performance shows a clear resemblance to the item mastery criteria but does not fully meet the criteria; and 2 = the child performs the item according to the criteria specified for mastery). Our results suggested that the intermediate score “1” was relevant to identifying children's performance aligned with the authors of PDMS-2; they suggested that the intermediate categories capture progressive change in children with motor delays (12). Our findings were not aligned with a previous study with Taiwanese children which suggested that most of the intermediate criteria provided less information about children's performance and were redundant for typically developing children, where the researchers suggest simplifying the items to dichotomous categories (15). The intermediate scores were necessary to the PDMS-2 capacity to discriminate different performance levels in our results.

The PDMS-2 showed good fit indices and the capacity to differentiate children's diverse motor skill performance levels for all subscales. However, it is essential to notice that the reflexes subscale had lower discrimination capacity due to the reduced number of items compared to the other subscales. It is an essential outcome of an instrument the discriminant capacity to distinguish typical and non-typical motor very early in the child's life; the early diagnosis provided support for early intervention and may impact children's development throughout life (5, 6). Consequently, the discriminating capacity of any instrument in the first years of life is an essential component for clinical and educational practice (35–37).

Another interesting result in this study was the unexpected patterns of response (higher infit or lower outfit from acceptable cutoffs points) to some items, reflexes (two items), stationary (one item), grasping (one item), and visual-motor integration (seven items); however, those items did not affect the PDMS-2 psychometrics and can be used to assess Brazilian children. Previously, a study with Taiwanese children also found unexpected results for stationary, grasping, and visual-motor integration subscales of the PDMS-2 (38). However, contrary to our results, the authors suggested that those items were not adequate to assess Taiwanese children with higher motor skills since the items were very easy and would only be suitable for assessing children with lower motor skills (36). The visual-motor grasp and integration subscales require fine motor attributes, and due to cultural differences, Taiwanese children may have more advanced manual dexterity than American children.

In advancing the previous study, the person-item map provided evidence for a hierarchical order in the PDMS-2 items that consider the child's motor performance and the difficulty of the items. Furthermore, the separation into distinct performance groups showed the ability of PDMS-2 to detect different levels of motor performance in all age groups. This combined information shows the sensitivity of PDMS-2 in detecting changes, crucial information for identifying infants and children who need further clinical support, and referral to a specific intervention. From understanding the difficulty levels of the items, it is possible to develop targeted activities and plan interventions in the short and long term. In advance, through this study, with the results found, it is possible to observe how children may go in a singular path from easier to more difficult items. The items' hierarchy should further be investigated in the light of maternal practices and children's motor experiences. Future studies could also undertake the challenge of examining items' difficulty and hierarchy for children with different disabilities.

The study has several limitations. First, the lack of previous studies examining item psychometrics restrains our capacity for comparisons. Second, our sample was composed mainly by typically developing children; investigating these goals in samples also composed of children with disabilities could provide different trends in the item's latent trail continuum. Third, our sample was composed of parents willing to participate and have their child assessed by professionals; these parents may have a concern about child development or may be aware of the importance of motor performance for the child's overall development. Although we are aware that most children's research is conducted with voluntary parents, we also need to recognize that it may present a bias to this research.

## Conclusion

The results observed in this study emphasize that the PDMS-2 is a reliable measure to identify motor changes in Brazilian children with different levels of performance in research and clinical and educational contexts. We found 11 maladjusted items; however, removing these items does not influence the PDMS-2 structure psychometric. Our results also showed that the addition of items with the middle level of challenge could be an option to compensate the scale discontinuity—for the effects of gaps between easy and very difficult items; may the addition of new items could strengthen the scale power to assess child development.

## Data Availability Statement

The original contributions presented in the study are included in the article/supplementary material, further inquiries can be directed to the corresponding author/s.

## Ethics Statement

The studies involving human participants were reviewed and approved by Federal University of Rio Grande do Sul Ethics Committee (n. 32071). Written informed consent to participate in this study was provided by the participants' legal guardian/next of kin.

## Author Contributions

LZ and NV wrote sections of the manuscript, contributed to conception and design of the study. LZ organized the database. All authors contributed to manuscript revision, read, and approved the submitted version.

## Funding

This work was funded by Coordenação de Aperfeiçoamento de Pessoal de Nível Superior (CAPES) and Conselho Nacional de Desenvolvimento Científico e Tecnológico (CNPq).

## Conflict of Interest

The authors declare that the research was conducted in the absence of any commercial or financial relationships that could be construed as a potential conflict of interest.

## Publisher's Note

All claims expressed in this article are solely those of the authors and do not necessarily represent those of their affiliated organizations, or those of the publisher, the editors and the reviewers. Any product that may be evaluated in this article, or claim that may be made by its manufacturer, is not guaranteed or endorsed by the publisher.
